# Bioinspired Interfacial Spontaneous Growth of ZnO Nanocatalysts onto Recycled Textiles as a Sustainable Approach for Water Purification

**DOI:** 10.1002/gch2.202200110

**Published:** 2022-11-14

**Authors:** Xi Wang, Yuan Wang, Menyan Nie, Stephen Cowling, Xiaogang Chen, Jian R. Lu, Xuqing Liu

**Affiliations:** ^1^ Department of Materials School of Natural Sciences Faculty of Science & Engineering The University of Manchester Manchester M13 9PL UK; ^2^ Institute for Materials Discovery Faculty of Maths & Physical Sciences University College London London WC1E 7JE UK; ^3^ Biological Physics Group Department of Physics and Astronomy School of Natural Sciences University of Manchester Oxford Road Manchester M13 9PL UK

**Keywords:** dyed water treatment, electroless deposition, polydopamine, waste textiles, zinc oxide

## Abstract

Zinc oxide, as a commonly used photocatalytic degradation of organic pollutants, typically shows limitations in wastewater treatment, such as aggregation and recycling problems caused by nanoscale dimensions and inappropriate substrates. Anchoring ZnO on substrates is a strategy to obtain stable catalytic performance. Particularly, natural fibers with hollow structures are an attractive alternative for ecological and economical ZnO loading templates, but depositing ZnO onto hollowed fiber surfaces presents a challenge. Here, a straightforward in situ growth method for producing nanostructured zinc oxide on cotton fibers from recycled garments is reported. The modified polydopamine on the fiber surface captures the catalyst required for in situ growth of ZnO and serves as a platform for spontaneous catalytic crystal growth on the fiber surface. The ZnO nanocrystals are uniformly dispersed on the outer and inner walls of cotton fibers, demonstrating excellent durability in wastewater treatments. Moreover, the photocatalytic performance of functional fibers is optimized by doping Ag nanoparticles to improve degradation efficiency. This can extend the prospect of further applications of developed ZnO/fibers in optoelectronics, spintronics, and provide inspiration for recycling and upgrading of used garments.

## Graphical abstract

Nanostructured zinc oxide with photocatalytic properties grows spontaneously and evenly on both outer and inner walls of hollowed fibers and exhibits excellent ultraviolet degradation of organic pollutants in sewage. The recycled cotton textiles are shown to be a sustainable platform for the in situ spontaneous growth of ZnO, after interfacial modification by nature‐inspired polydopamine.

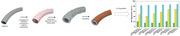

## 1 Introduction

Water pollution is one of the most serious environmental problems, and it is inextricably linked to both current and future generations’ health.^[^ ^1^ ^]^ It has been reported that over 100 000 different varieties and approximately 700 000 tons of synthetic dyes have been commercially applied worldwide, as main industrial contaminants eventually discharged into rivers or oceans.^[^ ^1^, ^2^ ^]^ Moreover, due to the growing industrial population, the lack of fresh drinking water is a worldwide problem.^[^ ^3^ ^]^ Therefore, removing or degrading the pollutants in wastewater is a priority in worldwide development.^[^ ^1^ ^]^

To solve these issues, a variety of wastewater treatment techniques are used, including ultraviolet (UV)‐catalysis, membrane filtration, reverse osmosis, electrocoagulation, and adsorption.^[^ ^2^, ^3^, ^4^ ^]^ Among these techniques, UV‐catalysis has been demonstrated to be one of the most energy‐efficient and cost‐effective.^[^ ^5^ ^]^ Ultraviolet light is referring to the electromagnetic spectrum of wavelength between 10 and 400 nm.^[^ ^6^, ^7^, ^8^ ^]^ Since UV light can be obtained from the sun or some other irradiation light source as a green energy resource, it has plenty of applications, such as photocatalysis, ultraviolet photoelectron spectroscopy, sterilization, disinfection, and optical sensors.^[^ ^4^, ^9^ ^]^ Generally, photocatalytic materials such as titanium dioxide (TiO_2_), ZnO, and tin dioxide, have been widely investigated.^[^ ^10^ ^]^ For instance, TiO_2_ is generally regarded as one of the best photocatalytic materials, because of its stable physical property and wide band.^[^ ^11^ ^]^ However, while compared with TiO_2_, zinc oxide has unique advantages including direct bandgap as well as high electron mobility.^[^ ^12^ ^]^ As an n‐type semiconductor with high redox potential, ZnO, is a superior material for photocatalysts. As previous research indicated, ZnO can absorb ultraviolet light in ultraviolet a (UVA) (320–400 nm) and ultraviolet b (UVB) (290–320 nm) range.^[^ ^13^ ^]^ This nontoxic, stable, and inexpensive semiconductor material, ZnO, has a broad bandgap of 3.37 eV and an excitation binding energy of 60 meV with excellent UV‐blocking, optical, UV‐catalytic, and antibacterial properties.^[^ ^6^, ^14^ ^]^ To reduce the effect of the relative wide bandgap and further extend the photocatalytic application of ZnO, doping with metal ions or nonmetal or rare earth elements is applied as an effective route for the photocatalysts with superior degradation performance.^[^ ^15^ ^]^

It has been reported that nanostructured ZnO can provide excellent solar photocatalytic properties.^[^ ^14a^ ^]^ However, due to the diffusion of pure ZnO nanomaterials into wastewater, the recycling of ZnO after UV‐catalysis brings a great challenge. Moreover, secondary pollution can be induced after the degradation. Therefore, supported photocatalysts are suggested to improve the practicability.^[^ ^16^ ^]^ A few polymers, such as poly(vinyl acetate) and poly(vinyl pyrrolidone) have been applied as support substrates to prepare ZnO based on templates.^[^ ^17^ ^]^ Particularly, to improve efficiency, a high specific area is demanded to increase the activation side. A bioinspired 3D polyurethane sponge construction provided a self‐standing skeleton with the interconnected macroporous structure for the in situ growth of ZnO nanorods.^[^ ^18^ ^]^ The hybrid composites exhibited a hierarchical structure to improve the environmental purification and energy conversion ability. Therefore, hollow structured materials are favored by the high‐specific‐area property. Notably, the continuously huge waste in the fast fashion industry has been widely criticized as well as its excessive consumption of resources. To reduce the environmental impacts, reusing and redeveloping the wasted garment would provide sustainable approaches to textile recycling. Hence, for natural materials, cotton, one of the most widely used fibers, has a unique hollow structure, which will be convenient to reuse from the waste garment providing a new source of fiber substrates, when a high surface area is required.^[^ ^19^ ^]^ Moreover, it is a natural template that can decrease pollution during the template synthesis process. However, the bonding between the inorganic active materials and the organic substrates would require further enhancement, which suggests that the reusability of the supported photocatalysts needs improvements. Inspired by mussels, the natural adhesion material, polydopamine, can provide spontaneous strong attachment on various platforms, including polymers, semiconductors, and ceramics.^[^ ^20^ ^]^ Hence, the polydopamine layer can be applied as a bonding interface between the inorganic active materials and the organic templates for later deposition of metal or semiconductor materials.^[^ ^21^ ^]^ However, the impact of interfacial bonding on the durability of ZnO deposited fiber in photocatalytic applications has limited investigations.

In this study, we are inspired by nature, and employ polydopamine as an interfacial active layer to deposit ZnO on recycled cotton fibers from the waste garments for the utilization of UV light. The natural entanglement had improved the attachment between the organic template and the semiconductors. Dye stuff on waste cotton fiber does not have a negative effect on the deposition of polydopamine but can theoretically promote the modification, because the conjugated structure of the dye stuff and the conjugation of polydopamine can be attracted by π–π stacking, which increases the surface binding between polydopamine and fibers. Meanwhile, nanostructured ZnO crystals were produced to provide effective photocatalytic performance with high activity. Furthermore, silver (Ag)‐doped ZnO cotton fibers would serve as an ideal platform with distortion of the crystal lattice to reduce the recombination of the photogenerated electron–hole pairs for enhancement of photoconversion efficiency and photodegradation activity. Hence, the as‐made ZnO hybrid photocatalysts can provide an effective UV degradation capacity. This method can also provide a strategy to fabricate functional textiles with various applications from waste garments. It can also provide a facile in situ growth strategy to fabricate functional textiles with various applications from waste garments, which can also provide great potential in environmental purification, energy storage, and wearable electronics area.

## 2 Results and Discussion

### 2.1 Polydopamine Modification of Cotton Fiber Substrates

**Scheme 1:**
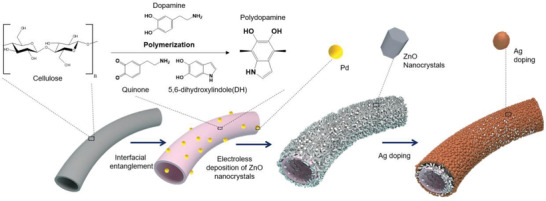
The fabrication process of hybrid ZnO photocatalyst loaded on cotton fibers for dyed water pollution treatment.

**Scheme** **1** illustrates the procedures of fabricating ZnO‐loaded functional fibers by electroless deposition method with polydopamine biomass as natural entanglements. Inspired by mussels, dopamine was polymerized and attached to the surface of fibers as a firm platform for the following electroless deposition reactions. Since polydopamine is also a biodegradable natural material, this approach can be applied to various biosubstrates for further investigations. Here, for example, we choose cotton fibers from waste garments as substrates to grow ZnO due to cotton occupied more than 50% garment materials market. In another hand, the reusability of the waste garments as substrates will contribute sustainable fashion and future lifestyle. In the experiment, polydopamine was coated on the fiber surfaces through the dip doping method in a tris buffer at room temperature for 24 h. Then, the specimens were rinsed in deionized water 3 times and dried in N_2_ gas steam.

**Figure 1:**
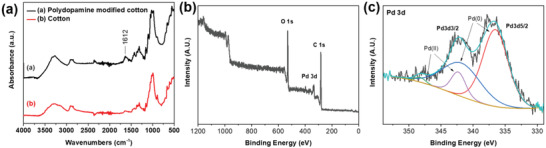
a) FTIR spectra of polydopamine coated cotton fibers and pristine cotton fibers. b) XPS spectrum of PDA and Pd modified cotton fibers cotton. XPS spectrum of c) Pd 3d for the PDA and Pd modified cotton sample.

Fourier transform infrared spectroscopy (FTIR) analysis indicates that polydopamine was successfully coated onto cotton fibers. The spectra of pure cotton fibers and cotton fibers coated with polydopamine are shown in **Figure** **1**. A peak at 1612 cm‐1 was observed, which can be attributed to the N‐H bending vibration within polydopamine's primary amine groups.^[^ ^22^ ^]^ Following polydopamine coating, the samples were submerged in an ammonium tetrachloropalladate aqueous solution for 1.5 h in a dark atmosphere. The polydopamine coating enhances the link between the organic substrates and the inorganic active materials, as the polydopamine primary amine group can chelate the inorganic active materials.

The spectra of pure cotton fibers and cotton fibers coated with polydopamine are shown in Figure 1. A peak at 1612 cm‐1 was observed, which can be attributed to the N‐H bending vibration within polydopamine's primary amine groups.^[^ ^22^ ^]^ Following polydopamine coating, the samples were submerged in an ammonium tetrachloropalladate aqueous solution for 1.5 h in a dark atmosphere. The XPS spectra (Figure 1b,c) show the existence of Pd 3d peak of modified cotton fibers cotton, suggesting the successful entrapment of Pd(0)/Pd^2+^ ions within the polydopamine interface. The polydopamine coating enhances the interface between the organic substrates and the inorganic active materials, as polydopamine's main amine group can chelate the Pd^2+^ ions (Figure 1b,c). Additionally, it is claimed that the catechol groups in polydopamine can decrease some of the Pd^2+^ ions to nanoparticles within the polydopamine coating layer. The Pd^2+^ ions were trapped within the polydopamine interface during this process, which will contribute to catalyzing the subsequent electroless deposition.

### 2.2 Characterization of ZnO Loaded Cotton Fibers

**Figure 2:**
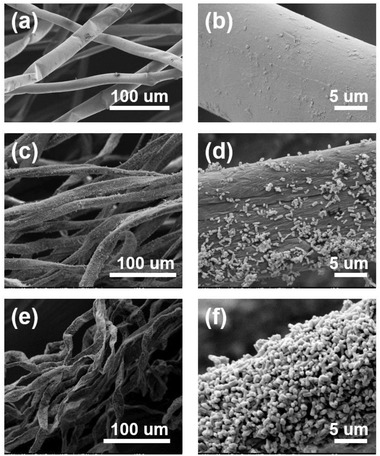
Morphology analysis of a,b) the pristine cotton fibers and ZnO loaded cotton fibers with c,d) 30 min and (e, f) 120 min prolonged time.

The SEM images of the pristine cotton fibers and the ZnO loaded cotton fibers with 5, 10, 30, 60, 90, and 120 min of deposition time are present in **Figure** **2** and Figure S1 (Supporting Information). Compared with pristine cotton fibers, the nanostructured ZnO crystals were distributed on the surface of fibers as the electroless deposition time increased. The SEM images (Figure S2, Supporting Information) show the fiber surface morphology with no dopamine pretreatment after the ZnO deposition process. The uneven spread of ZnO on the fibers suggested that dopamine offered an important platform for stable electroless deposition reactions. After 30 min of the deposition process, the 850 nm nanorod ZnO crystals started to attach to the surface of cotton fiber substrates. Then, a uniform layer of ZnO nanorods was observed on the substrate surface and the crystal size of ZnO became larger, around 950 nm, after 120 min of the deposition process. The dimethylamine borane (DMAB) solution was used as a reductant in this electroless deposition, which can improve the reduction of NO_3_^−^ ions to raise the pH. The local pH increase by the reduction reaction is a key factor that can lead to the precipitation of Zn ions in this electroless deposition process. To further illustrate the ZnO deposition on the substrates, elemental mapping for SEM images is shown in Figure S3 (Supporting Information). Under the strong carbon and oxygen signal from natural fibers, the zinc mapping image has proved that ZnO nanocrystals were widely spread over the fiber substrates.

**Figure 3:**
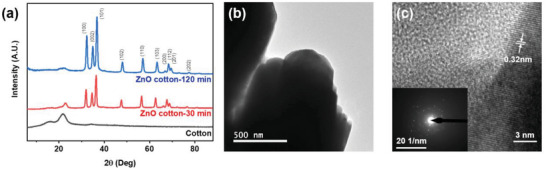
a) XRD spectra of ZnO cotton‐30 min, ZnO cotton‐120 min, and pristine cotton fibers. b) TEM image of a nanocrystal cluster of ZnO (120 min) and c) HRTEM image of the ZnO nanocrystal (120 min) with the SAED image inserted.

The XRD spectra of ZnO‐loaded cotton fibers and pristine cotton fibers were shown in **Figure** **3**a, which revealed an XRD pattern of pure cotton and ZnO indexed to the hexagonal wurtzite ZnO crystals (PDF‐01‐080‐6503). The two intense diffraction peaks at 16° and 22.5° correspond to typical cellulose (110) and (200) crystal faces.^[^ ^23^ ^]^ Moreover, both ZnO‐loaded fibers have three main diffraction peaks at 33°, 36°, and 38° corresponding to (100), (002), and (101) planes of zinc oxide with the hexagonal wurtzite crystal structure.^[^ ^24^ ^]^ When the reaction time increased, the XRD peaks of zinc oxide nanomaterial were enhanced and became higher and broader. The average sizes of ZnO crystallite loaded on fibers increased when ZnO deposition time was extended. Therefore, it indicated that prolonged time could assist with the control of ZnO crystalline growth with different sizes.

In Figure 3b, the TEM image illustrates a nanocrystal cluster of ZnO, and the SAED image also suggests the polycrystalline property. The cross‐section diameter size of a single ZnO nanocrystal is around 500 nm. The HRTEM image (Figure 3c) suggests the (101) atomic plane spacing around 0.32 nm in the hexagonal‐wurtzite‐structured ZnO crystals. The inserted corresponding selected area electron diffraction (SAED) pattern indicated a certain number of small ZnO crystals. The TEM results suggested the successful deposition of ZnO over the natural cotton fibers.

**Figure 4:**
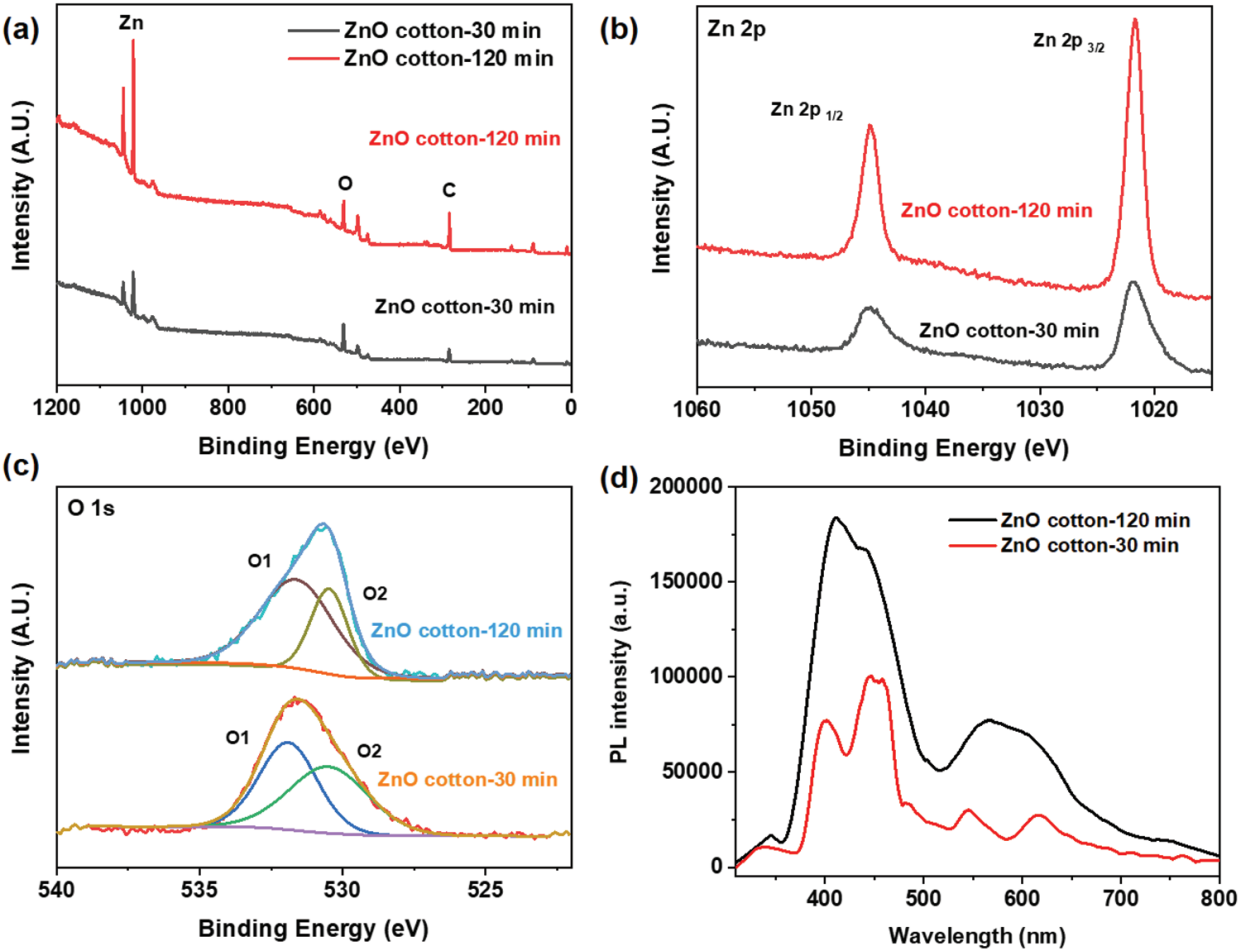
a) The survey XPS spectra of ZnO loaded cotton fibers with 30 and 120 min prolonged time. b) The Zn 2p spectrum of ZnO cotton‐30 min and ZnO cotton‐120 min, and c) O 1s spectrum of ZnO cotton‐30 min and ZnO cotton‐120 min. d) Photoluminescence (PL) spectrum of ZnO cotton‐30 min and ZnO cotton‐120 min.

To investigate the elemental compositions and chemical states, the X‐ray photoelectron spectroscopy (XPS) tests of ZnO cotton‐30 min and ZnO cotton‐120 min were conducted (**Figure** **4**). The XPS spectra of ZnO cotton‐30 min and ZnO cotton‐120 min show the typical zinc and oxygen peaks. The high‐resolution XPS spectrum of Zn 2p shows the peaks for 2p1/2 and 2p3/2. Meanwhile, the O1s peak was fitted with two Gaussian curves centered at 532.15 and 529.25 eV. In Figure 4c, the two deconvoluted peaks can be corresponding to the O^2−^ state from the oxygen defects or vacancies (O1 peak) and the O^2−^ state from the ZnO lattice oxygen (O2 peak), respectively. Compared with ZnO cotton‐30 min, the relative intensity of O1 to O2 peak of ZnO cotton‐120 min is increased, suggesting the increase in the concentration of oxygen defects or vacancies.^[^ ^25^ ^]^ Meanwhile, the intensity of the O2 peak indicates a stable metallic character from the ZnO lattice. As the oxygen vacancy defects can provide the downshift of the valence band, it would have a stronger water oxidation capability to generate the active hydroxyl radical species for better degradation performance.^[^ ^26^ ^]^ Figure 4d shows the photoluminescence (PL) spectra of the as‐prepared ZnO cotton‐30 min and ZnO cotton‐120 min. The sharp emission at 398 nm represents the intrinsic emission from ZnO, while the emission at 500–600 nm is related to the wide band defects, which can be ascribed to ionized surface oxygen vacancies.^[^ ^27^ ^]^ The ZnO cotton‐20 min shows the peaks at 398 nm and 500–600 nm with higher intensity than that of ZnO cotton‐30 min, suggesting a more complete ZnO crystal structure with abundant oxygen vacancies or defects. It would decrease the recombination of photogenerated electrons‐hole pairs to improving degradation performance.

### 2.3 Photodegradation Test for Water Treatment

The Ultraviolet‐visible spectrophotometer was applied to demonstrate the degradation efficiency of the methylene blue. The UV absorption spectra had peaks located at 663 nm, 292 nm, and 246 nm index to the characteristic peaks of methylene blue. A plausible MB photodegradation pathway was shown in Figure S4 (Supporting Information). The standard concentration curve was applied to determine the remaining methylene solution after different UV irradiation times. The efficiency of the photocatalyst is calculated according to this formula (Equation 1)

(1) $$\begin{array}{c} \text{Degradation}\;\text{rate}=\frac{C0-Ct}{C0}\times 100\% \end{array}$$

**Figure 5:**
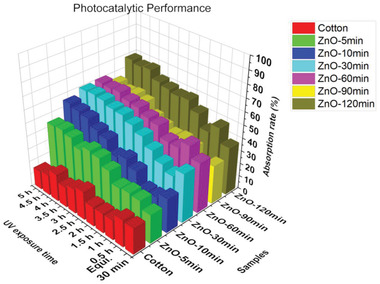
Photocatalyst performance of pristine cotton and ZnO loaded cotton fibers with 5, 10, 30, 60, 90, and 120 min prolonged time.

**Figure 6:**
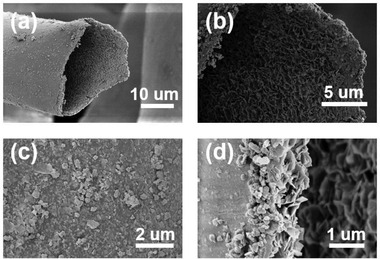
Morphology analysis of a) lumen structure of cotton fibers containing ZnO nanocrystals on the b) inner wall, c) the outer wall, and d) edge of cotton fibers after 5 h photodegradation tests and 5 laundry cycles.

C0 is the initial methylene blue solution concentration value, and Ct is the methylene blue solution concentration value after t min of the UV irradiation process. **Figure** **5** shows that the 120 min ZnO photocatalyst performed the best degradation efficiency. As the inner region of cotton fibers contains a lumen structure (shown in **Figure** **6**), ZnO nanocrystals were also discovered loaded on the inner wall of fibers, which can increase the active site of functional materials to improve the final application. For all the photocatalyst samples, the UV degradation efficiency can reach over 40% after 5 h UV irradiation. Compared with the pure cotton fibers which only have 15% of absorption efficiency, the 120 min ZnO photocatalyst can achieve almost 70% of degradation efficiency after a 5 h UV exposure process. It is worth noting that the ZnO nanocrystals have suffered some corrosion during the UV degradation process and 5 times washing cycles, as shown in Figure 6 and Figure S5 (Supporting Information). The morphology of ZnO nanocrystals disassembled on the outer walls of fibers (Figure 6) could be due to the photo corrosion and the physical friction effect. Particularly, the XRD patterns in Figure S5 (Supporting Information) also suggest that the wurtzite‐type crystal structure remained but with some damage even after 15 laundering cycles. The interfacial bonding between the natural fiber substrate and ZnO provides firm support for the stability of the deposition. Moreover, we applied the Instron tensile test machine to stretch the ZnO cotton until breaking and illustrate its cross‐section structures after UV irradiation (Figure 6). The unique hollow structure feature of the fiber substrate could also provide more deposition sites and more protection for the ZnO loadings on the inner walls of fibers. It indicated the good durability of ZnO cotton fibers during the UV degradation process and laundering cycles.

### 2.4 Improvement of Photodegradation Efficiency by the Doping Method

**Figure 7:**
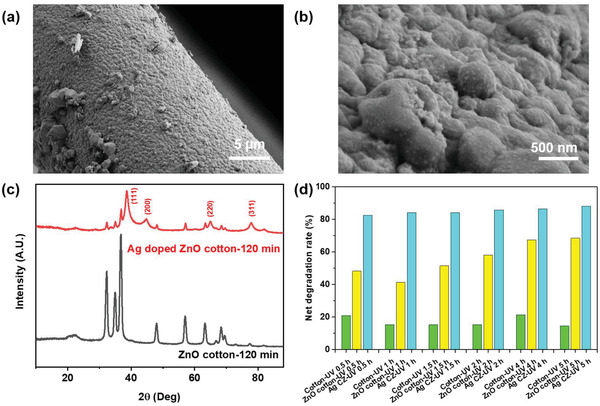
Surface morphology analysis of a,b) Ag‐doped ZnO cotton fibers; crystal structure analysis of c) pristine cotton fibers, ZnO loaded cotton fibers, and Ag‐doped ZnO cotton fibers. d) Absorption rate of MB by cotton and net degradation rate of MB treated by ZnO‐cotton 120 min and Ag‐doped ZnO fibers with different prolonged time after 0.5, 1, 1.5, 2, 4, 5 h UV irradiation process.

**Figure 8:**
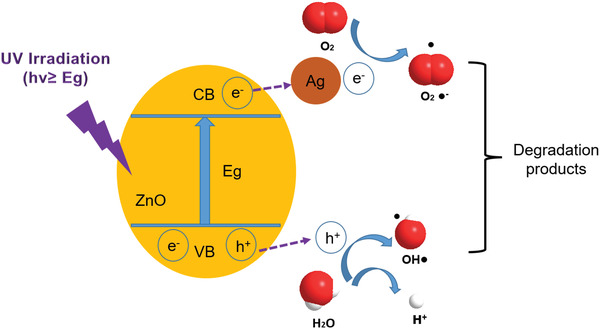
Mechanism diagram of Ag‐doped ZnO for photodegradation process under UV irradiation.

Transition metal (Ag) was also chosen as a deposition material for bicomposite. Ag nanoparticles (0.8 wt.%) with an average diameter of 50 nm covered over ZnO on fiber surface in **Figure** **7**a,b. The combined XRD patterns of ZnO‐loaded cotton fibers and Ag‐ZnO bi‐composite deposited cotton fibers are shown in Figure 7c. Four typical diffraction peaks are observed corresponding to diffraction planes (111), (200), (220), and (311).^[^ ^28^ ^]^ Especially, the diffraction peak at 38.03° in the heterostructure of Ag‐ZnO bi‐composite is attributed to the (111) plane of face‐cantered cubic (fcc) Ag, according to the standard diffraction data (ICDD 4–0783).^[^ ^29^ ^]^ Further, the mean crystallite size of Ag nanoparticles is estimated to be 5.25 nm calculated by the Scherrer formula. Upon Ag deposition, no appreciable change is observed for ZnO, which confirms that the Ag nanoparticles on zinc oxide‐loaded fibers show little influence on the crystallinity of ZnO. The net UV degradation performance of Ag‐ZnO bi‐composite supported UV degradation catalysts were all over 80% after 5 h UV irradiation in Figure 7d and Figure S6 (Supporting Information). Particularly, the net degradation rate of ZnO cotton after the initial 0.5‐h UV irradiation has increased by nearly 90% after the Ag doping process. Moreover, the Ag‐doped ZnO cotton confirms the enhancement of photocatalytic efficiency when compared with other photocatalysts, including 2Ag/ZnO (65% of MB degradation rate for 90 min visible irradiation), ZnO:Ag 5%/VL (45% of MB rate for 140 min visible irradiation), 0.1Cu/ZnO (19% of Rhodamine B (RhB) degradation rate for 120 min visible irradiation), and 1.5% Fe‐doped ZnO nanofibers (88% of MB degradation rate for 360 min UV irradiation).^[^ ^30^ ^]^ Figure S7a (Supporting Information) shows the UV–vis absorption spectra of ZnO cotton‐120 min and Ag‐doped ZnO cotton‐120 min. The Ag‐doped ZnO cotton‐120 min shows a strong absorption peak at a wavelength below 400 nm. Meanwhile, the band gap energies of ZnO cotton‐120 min and Ag‐doped ZnO cotton‐120 minis were measured via the extrapolation of the linear part of the graph at the Kubelka‐Munk function versus photon energy shown in Figure S7b (Supporting Information). The band gap energy of Ag‐doped ZnO cotton‐120 min is 3.23 eV, while that of ZnO cotton‐120 min is 3.29 eV. The decrease in bandgap energy shows the enhancement of the light absorption range, which can support harvesting more photons due to the Ag elements. When metal deposits on the surface of the semi‐conductor, the electrons are intended to transfer from the higher Fermi level of semi‐conductor to the lower Fermi level of metal until reaching the same Fermi levels and forming a Schottky barrier, which can effectively capture photogenerated electrons.^[^ ^31^ ^]^ Therefore, the UV degradation efficiency can be improved by promoting the separation of photogenerated electron–hole pairs (**Figure** **8**).^[^ ^32^ ^]^

## 3 Conclusions

The nanostructured ZnO with photocatalytic properties grows spontaneously on the surface of the fibers and exhibits excellent UV degradation properties of organic matter in sewage. Nature‐inspired polydopamine was used to modify the surface of hollow fibers and provided a platform for the in situ spontaneous catalytic growth of ZnO on fiber surfaces. The ZnO nanocrystals were deposited on both the inner and outer walls of cotton fibers, which can improve the wastewater degradation process to around 70% after 5 h UV irradiation. Moreover, after the Ag‐doping process, the net UV degradation performance of Ag‐ZnO bi‐composites has achieved over 80% of the dye pollutant. It demonstrates that cotton fibers can provide economic and ecologic platforms for ZnO loadings as photocatalysts for polluted water treatment and even extend further potential applications to realize sustainable use of ZnO photocatalyst.

## 4 Experimental Section

### Materials

Tris (hydroxymethyl) aminomethane, dopamine hydrochloride, dimethylamine borane, (DMAB), and ammonium tetrachloropalladate were purchased from Sigma‐Aldrich (U.K.). Zinc nitrate hexahydrate and methylene blue were purchased from Thermo Fisher Scientific (U.K.). Cotton fibers used in this research were obtained from used waste garments and washed with water and soapsuds before reusing to remove the remaining impurities from wearing. The fabrics were dried at room temperature for 24 h and then cut into 1 cm^2^ piece and further torn into bundles of fibers. Distilled water (DI water) was used for all solutions. All chemicals were used as received.

### Surface Modification of Cotton Fibers

3 g of cotton fibers were immersed into 0.005 mol L^−1^ tris(hydroxymethyl) aminomethane buffer solution with 2 g L^−1^ of dopamine for 24 h. To anchor Pd ions on the fiber before loading of ZnO, those specimens were immersed in a 5 × 10^−3^ m ammonium tetrachloropalladate aqueous solution for 30 min. Then the fibers were rinsed in ethanol solution to remove organic impurities. Afterward, the samples were rinsed in DI water and dried at room temperature. To deposit ZnO, 30 × 10^−3^ m zinc nitrate hexahydrate and 30 × 10^−3^ m DMAB were dissolved in deionized water. Then the fibers and solution were mixed in an autoclave and placed in an oven at 90 °C for 5, 10, 30, 60, 90, and 120 min, respectively. These samples were denoted as ZnO cotton‐X min, in which X represents the prolonged time. Then, the samples were rinsed in deionized water and dried at 40 °C for 12 h. The previous small molecular dyes have little impact on the deposition process, as all fibers have been evenly covered with a layer of white ZnO nanocrystals.

### Depositing Silver on ZnO‐Loaded Fibers

To deposit silver, 10 g ZnO loaded fiber samples with different prolonged times were put into a 5% silver nitrate water solution heated at 100 °C for an hour. Silver nitrate (AgNO_3_) was decomposed into silver by heating treatment. Then the fibers were rinsed with DI water and dried at room temperature.

### Characterization of ZnO‐Loaded Cotton Fibers

Morphology of ZnO deposited cotton fibers was observed by scanning electron microscopy (SEM) through a Zeiss Ultra 55 microscope with an accelerating voltage of 3 kV and by transmission electron microscope (TEM) through an FEI Tecnai G2 20 (LaB6) microscope. The crystal structure of ZnO was investigated by X‐ray diffraction (XRD) with a PANalytical (Phillips) X'Pert Pro X‐ray diffractometer. The XRD patterns were tested at an accelerating voltage of 40 kV and anode current of 40 mA with 2θ range from 4° to 90° and a copper tube X‐ray source (λ = 0.154 nm). The changes in the organic functional group were measured by a Fourier Transform Infrared Spectroscopy (FTIR, Bruker Vertex 80). To account for depositing Ag, the XRD patterns and Raman spectra were measured respectively, in which Raman spectra were recorded with a Renishaw RM1000 – 514 nm Raman system with 1 µm the laser spot size and below 10 mW the power at samples. X‐ray Photoelectron Spectroscopy (XPS) was conducted using an Axis Ultra Hybrid spectrometer (Kratos Analytical, Manchester, United Kingdom) with monochromated Al Kα radiation (1486.6 eV, 10 mA emission at 150 W, spot size 300 × 700 µm) under a base vacuum pressure of ≈5 × 10^−9^ mbar. Charge neutralization was achieved via a filament. Binding energy scale calibration was performed with C—C in the C 1s photoelectron peak at 285 eV. Analysis and curve fitting was performed using Voigt‐approximation peaks through CasaXPS software.

### Photodegradation and Durability Test for Water Treatment

The photodegradation test was conducted under a 36 W ultraviolet lamp. 0.1 × 10^−3^ m methylene blue aqueous solution was mixed with 0.05 g of ZnO loaded cotton fibers in transparent glass bottles, which were 25 cm away from the light source in a chamber. The pristine cotton fiber was used as a contrast group. Then all the samples are placed in a dark environment for half an hour to reach a deposition balance. To demonstrate the photocatalysis degradation property, an ultraviolet–visible spectrophotometer was applied to measure the remaining methylene blue in liquid samples after 5 h of UV irradiation. To determine the durability of ZnO deposition on the fiber substrates, the ZnO‐loaded cotton samples were washed up to 15 cycles with an ECE Formulation Standard phosphor‐free laundry detergent (enzyme‐free). Then, the morphologies of ZnO‐loaded cotton samples after washing cycles were investigated by SEM and XRD experiments to investigate their stability and durability. Additionally, the Ag‐doped specimens were prepared through the same procedures to reveal the UV‐degradation performance after dark environment pretreatment.

## Conflict of Interest

The authors declare no conflict of interest.

## Supporting information

Supporting Information

## Data Availability

The data that support the findings of this study are available in the supplementary material of this article.
