# Supplementary material for: Bioinspired Interfacial Spontaneous Growth of ZnO Nanocatalysts onto Recycled Textiles as a Sustainable Approach for Water Purification
Source: Glob Chall. 2022 Nov 14;7(2):2200110. doi: 10.1002/gch2.202200110 (PMC9900724; doi:10.1002/gch2.202200110)
Supplement: Supplementary file 1 — Supporting Information [file GCH2-7-2200110-s001.pdf]

Global Challenges
Open Access

Supporting Information

for *Global Challenges,* DOI: 10.1002/gch2.202200110

Bioinspired Interfacial Spontaneous Growth of ZnO Nanocatalysts onto Recycled Textiles as a Sustainable Approach for Water Purification

*Xi Wang, Yuan Wang, Menyan Nie, Stephen Cowling, Xiaogang Chen, Jian R. Lu, and Xuqing Liu**

Supporting Information

Bioinspired Interfacial Spontaneous Growth of ZnO Nano catalysts onto Recycled Textiles as a Sustainable Approach for Water Purification

*Xi Wang, Yuan Wang, Menyan Nie, Stephen Cowling, Xiaogang Chen, Jian R. Lu, and Xuqing Liu**

X. Wang, S. Cowling, X. Chen, and X. Liu
Department of Materials, School of Natural Sciences, Faculty of Science & Engineering, The University of Manchester, Manchester, M13 9PL, UK.
Y. Wang and M. Nie
Institute for Materials Discovery, Faculty of Maths & Physical Sciences, University College London.
J. Lu
Biological Physics Group, Department of Physics and Astronomy, School of Natural Sciences, University of Manchester, Oxford Road, Manchester M13 9PL, UK.

E-mail: xuqing.liu@manchester.ac.uk

Keywords: natural fibers, zinc oxide, polydopamine, electroless deposition, photodegradation, dyed water treatment

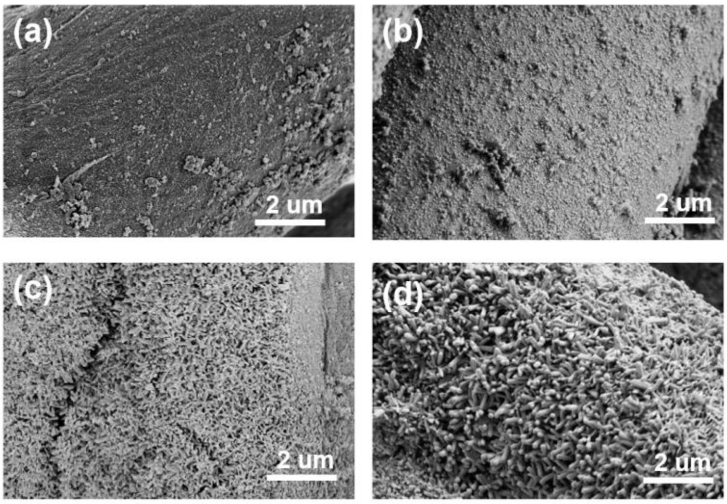

Figure S1. Morphology analysis of the ZnO loaded cotton fibers with (a) 5 mins, (b) 10 mins, (c) 60 mins, and (d) 90 mins prolonged time.

The morphologies of ZnO cotton-5min, 10 min, 60 min, and 90 min were shown in **Figure S1** to illustrate the growth process of ZnO electroless deposition on cotton fibers.

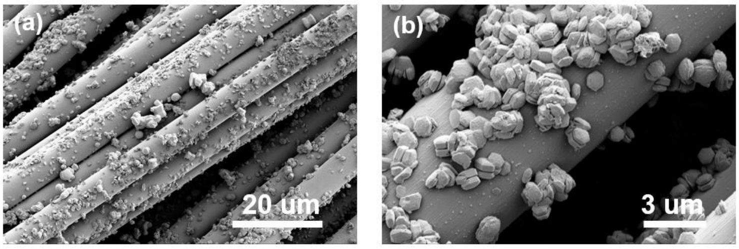

Figure S2. (a-b) SEM image for morphology analysis of the ZnO loaded cotton fibers without polydopamine pretreatment.

The SEM images (**Figure S2**) show the fiber surface morphology with no dopamine pretreatment after the ZnO deposition process. The uneven spread of ZnO on the fibers suggested that dopamine offered an important platform for stable electroless deposition reactions.

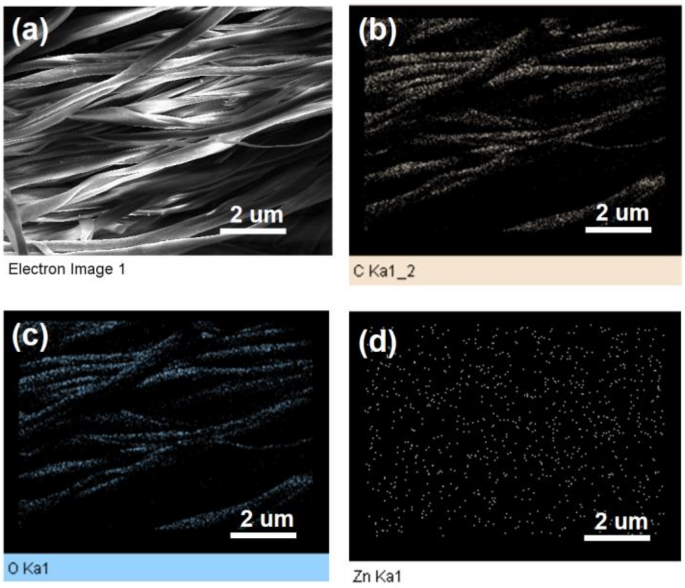

Figure S3. EDX analysis of the ZnO loaded cotton fibres

To further illustrate the ZnO deposition on the substrates, elemental mapping for SEM images is shown in **Figure S3**. Under the strong carbon and oxygen signal from natural fibres, the zinc mapping image has proved that ZnO nanocrystals were widely spread over the fibre substrates

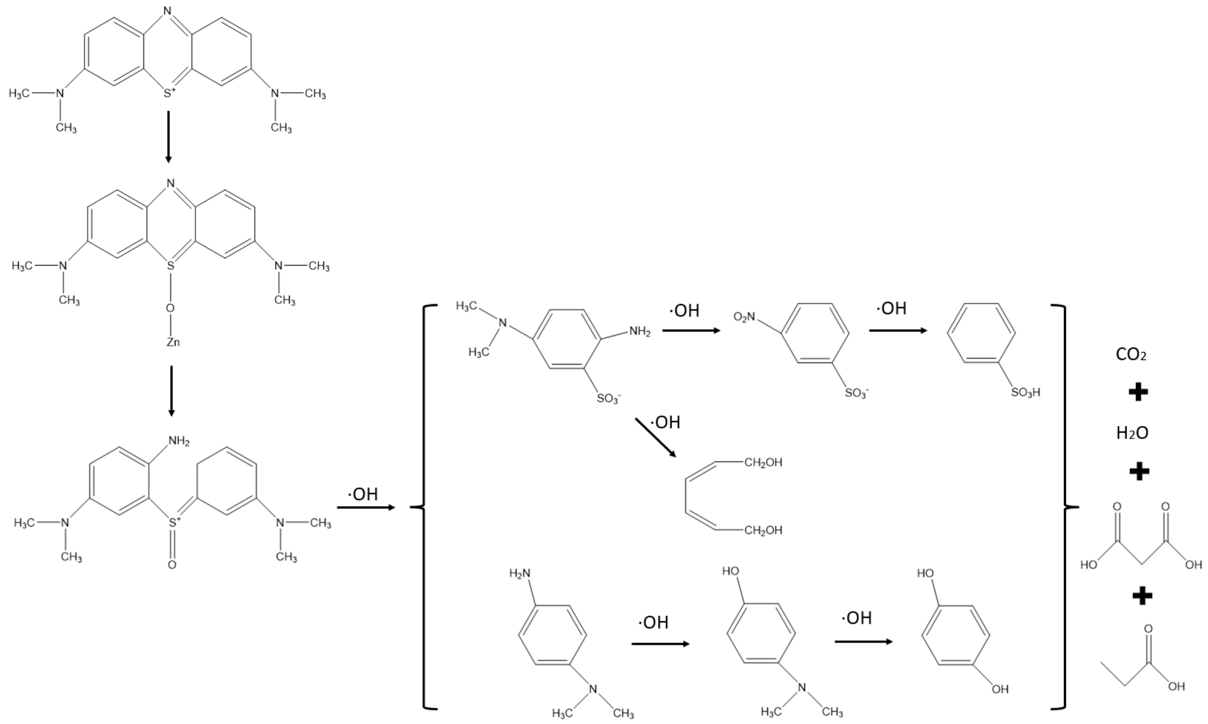

Figure S4. A plausible MB photodegradation pathway.

A plausible MB photodegradation pathway was displayed in **Figure S4**.[1]

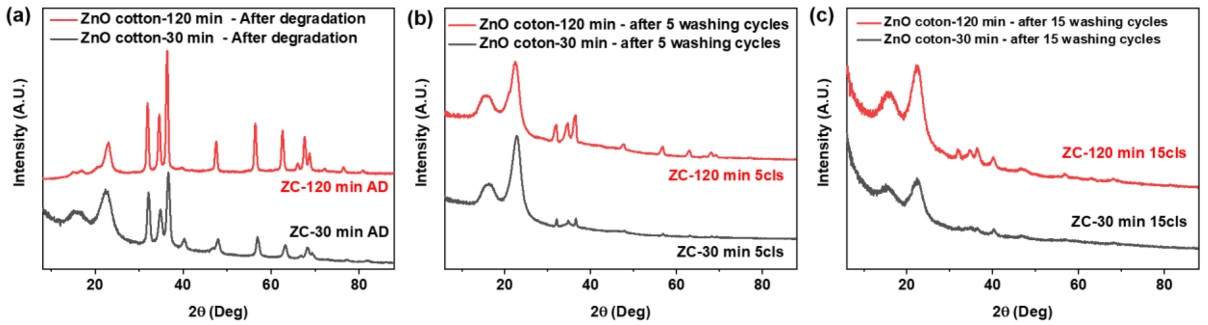

Figure S5. XRD patterns of (a) cotton fibers contain ZnO nanocrystals after photodegradation tests, cotton fibers contain ZnO nanocrystals after (b) 5 laundering cycles, and (c) 15 laundering cycles.

The XRD patterns of ZnO cotton-30 min and ZnO cotton-120 min after photodegradation tests, 5 laundering cycles, and 15 laundering cycles were shown in **Figure S5**. The ZnO characteristic peaks suggest the remaining ZnO loaded on the cotton substrates.

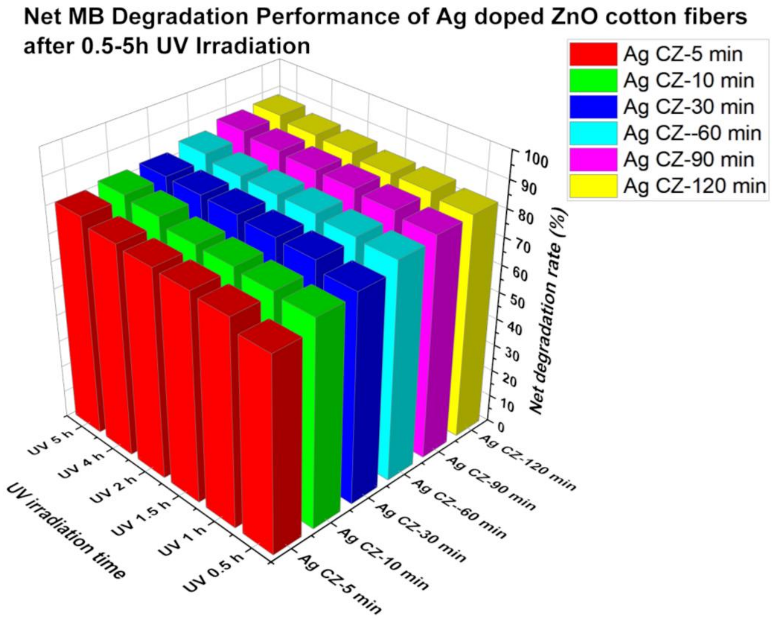

Figure S6. Degradation rate of MB treated by Ag-doped ZnO fibers with different prolonged times after 0.5, 1, 1.5, 2, 4, 5 hours UV irradiation process.

The net UV degradation performance of Ag-ZnO bi-composite supported UV degradation catalysts were all over 80 % after 5-hour UV irradiation in **Figure S6**.

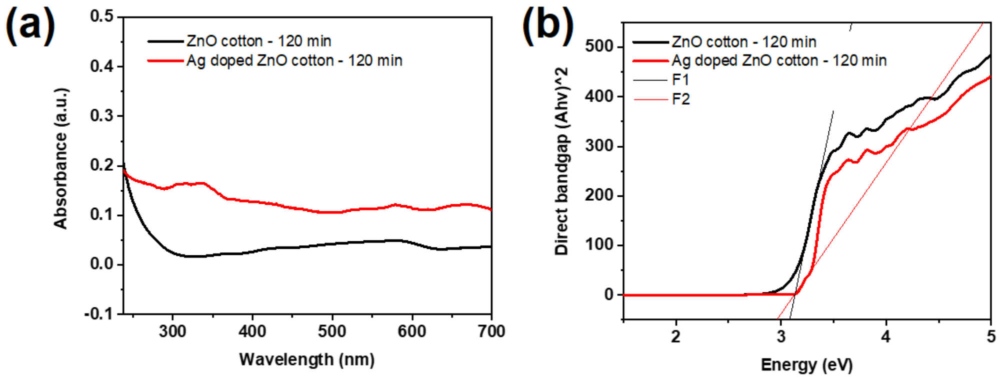

Figure S7. (a) UV-vis absorption spectra and (b) Kubelka-Munk function versus energy plots of ZnO cotton-120 min and Ag-doped ZnO cotton -120 min.

Figure S7(a) shows the UV-vis absorption spectra of ZnO cotton-120 min and Ag-doped ZnO cotton-120 min. The Ag-doped ZnO cotton-120 min shows a strong absorption peak at a wavelength below 400 nm. Meanwhile, the band gap energies of ZnO cotton-120 min and Ag-doped ZnO cotton-120 minis were measured via the extrapolation of the linear part of the graph at the Kubelka-Munk function versus photon energy shown in Figure S6(b).

References

[1] X. Wang, S. Xu, E. Chalmers, X. Chen, Y. Liu, X. Liu, *ACS Applied Materials & Interfaces* **2022**, 14, 10769.
